# Identification of a novel prophage regulator in *Escherichia coli* controlling the expression of type III secretion

**DOI:** 10.1111/j.1365-2958.2011.07927.x

**Published:** 2011-12-09

**Authors:** Allen F Flockhart, Jai J Tree, Xuefang Xu, Maryia Karpiyevich, Sean P McAteer, Ronen Rosenblum, Darren J Shaw, Christopher J Low, Angus Best, Victor Gannon, Chad Laing, Kenan C Murphy, John M Leong, Thamarai Schneiders, Roberto La Ragione, David L Gally

**Affiliations:** 1 Immunity and Infection Division, The Roslin Institute and R(D)SVS, University of EdinburghEdinburgh EH25 9RG, UK; 2 Centre for infection and Immunity, 4th Floor Medical Biology Centre, School of Medicine, Dentistry and Biomedical Sciences, Queen's University BelfastBelfast BT9 7BL, UK; 3 Division of Veterinary Clinical Sciences, Royal (Dick) School of Veterinary Studies, The University of Edinburgh, Easter Bush CampusRoslin, Midlothian EH25 9RG, UK; 4 Department of Food and Environmental Safety, Veterinary Laboratories AgencyWoodham Lane, New Haw, Addlestone, Surrey, KT15 3NB, UK; 5 Laboratory for Foodborne Zoonoses, Public Health Agency of Canada1st floor, C.F.I.A. Building, Lethbridge, AB T1J 3Z4, Canada; 6 Department of Molecular Genetics and Microbiology, University of Massachusetts Medical SchoolWorcester, MA 01655, USA; 7 Faculty of Health and Medical Sciences, University of SurreyGuildford, Surrey, GU2 7XH, UK

## Abstract

This study has identified horizontally acquired genomic regions of enterohaemorrhagic *Escherichia coli* O157:H7 that regulate expression of the type III secretion (T3S) system encoded by the locus of enterocyte effacement (LEE). Deletion of O-island 51, a 14.93 kb cryptic prophage (CP-933C), resulted in a reduction in LEE expression and T3S. The deletion also had a reduced capacity to attach to epithelial cells and significantly reduced *E. coli* O157 excretion levels from sheep. Further characterization of O-island 51 identified a novel positive regulator of the LEE, encoded by *ecs1581* in the *E. coli* O157:H7 strain Sakai genome and present but not annotated in the *E. coli* strain EDL933 sequence. Functionally important residues of ECs1581 were identified based on phenotypic variants present in sequenced *E. coli* strains and the regulator was termed RgdR based on a motif demonstrated to be important for stimulation of gene expression. While RgdR activated expression from the LEE1 promoter in the presence or absence of the LEE-encoded regulator (Ler), RgdR stimulation of T3S required *ler* and Ler autoregulation. RgdR also controlled the expression of other phenotypes, including motility, indicating that this new family of regulators may have a more global role in *E. coli* gene expression.

## Introduction

*Escherichia coli* strains are usually present in the flora of mammalian gastrointestinal (GI) tracts and many are considered non-pathogenic. However, some strains are associated with serious intestinal and extra-intestinal infections. The main differences among strains of these different pathotypes can be attributed to the acquisition of genetic information from mobile genetic elements, in particular bacteriophage (Kaper *et al*., 2004). Bacteriophage integration and recombination not only leads to phage evolution, but is a key driver in the acquisition of virulence attributes in enteric bacteria, and of the complex regulatory networks that control their expression. Enterohaemorrhagic *E. coli* (EHEC) contain prophage-encoded Shiga toxins and are associated with severe GI and systemic disease in humans (Nataro and Kaper, 1998; Karmali, 2004). Ruminants are considered to be the most important reservoirs for EHEC, particularly cattle and sheep which shed the organism in their faeces (La Ragione *et al*., 2009). The predominant pathogenic serotype in North America, parts of Asia and the UK is O157:H7 (Armstrong *et al*., 1996; Besser *et al*., 1999; Caprioli *et al*., 2005).

Studies on EHEC O157:H7 have shown that the genotypic diversity among different strains is largely attributable to bacteriophage-related DNA fragments (Ohnishi and Hayashi, 2002; Zhang *et al*., 2007; Asadulghani *et al*., 2009). In EHEC O157:H7 strain EDL933, these horizontally acquired elements have been termed O-islands (OIs) and include both fully functional and cryptic prophages (Perna *et al*., 2001). The locus of enterocyte effacement (LEE) encodes a type III secretion (T3S) system, an essential virulence factor for EHEC that allows the direct injection of bacterial effector proteins into host cells to subvert host cell signalling pathways and promote bacterial attachment (Kenny *et al*., 1997; Hemrajani *et al*., 2010; Newton *et al*., 2010). While T3S has been shown to be critical for EHEC O157:H7 colonization in the ruminant host (Naylor *et al*., 2005), levels of T3S can vary significantly among different EHEC isolates (Son *et al*., 2002; Roe *et al*., 2003; Rashid *et al*., 2006; Yang *et al*., 2009). Such variation in T3S regulation may have important implications for bacterial persistence and shedding in the bovine host and for human infection (Chase-Topping *et al*., 2008). Recent studies comparing EHEC O157:H7 genome sequences have identified three lineages of the organism (Kim *et al*., 1999; Zhang *et al*., 2007). While all lineages can be isolated from cattle, lineage I (LI) and lineage I/II (LI/II) strains are more frequently associated with human clinical disease than lineage II (LII) strains. Further, EHEC O157:H7 strains associated with outbreaks of severe illness in the USA have been shown to belong to a single subtype of LI/II, designated SNP clade 8 (Manning *et al*., 2008; Laing *et al*., 2009). Several studies have attempted to understand the genetic basis for the apparent differences in the epidemiology and virulence among EHEC O157:H7 genotypes, and recent studies have shown that LI and LI/II strains produce higher levels of Shiga toxins than bovine-associated LII strains (Zhang *et al*., 2010). Similarly, hyper-virulent SNP clade 8 strains have been shown to have higher levels of LEE expression than EHEC O157:H7 strain Sakai from SNP clade 2 (Abu-Ali *et al*., 2010).

A large number of factors have been shown to influence expression of LEE in both enteropathogenic *E. coli* (EPEC) and EHEC (Tree *et al*., 2009; Kendall *et al*., 2010). In both pathotypes, the LEE-encoded regulator (Ler) is expressed from the first LEE operon (LEE1) and is required to activate expression of all five main LEE operons (LEE1–5) (Elliott *et al*., 2000; Sharma and Zuerner, 2004). However, at higher concentrations, Ler is considered to be repressive at LEE1 (Berdichevsky *et al*., 2005; Yerushalmi *et al*., 2008). To date, most of the genetic and environmental control of T3S has been shown to occur through LEE1 and Ler activation, including an activator and repressor combination within the LEE, GrlA and GrlR, which are involved in the reciprocal regulation of LEE and flagella expression (Barba *et al*., 2005; Iyoda *et al*., 2006; Huang and Syu, 2008; Jimenez *et al*., 2010; Islam *et al*., 2011;). In EPEC, LEE expression is thought to be positively regulated by the plasmid-encoded regulator C (PerC) locus and a series of PerC homologue (Pch) proteins; encoded by *pchA–E* and *pchX* in EHEC O157:H7. These genes are usually associated with cryptic prophage regions encoding effector proteins that are exported by the T3S system and the Pch regulators co-ordinate the expression of these horizontally acquired effectors with the LEE-encoded T3S system (Iyoda and Watanabe, 2004; Porter *et al*., 2005; Abe *et al*. 2008; Yang *et al*., 2009).

The aim of the present study was to identify OIs of EHEC O157:H7 that cross-talk with the T3S system. An OI-51 deletion mutant was shown to have lower levels of LEE expression *in vitro* and subsequent testing demonstrated that this genomic island contributes to ruminant colonization and persistence. A novel regulator, termed RgdR, was identified on OI-51 and shown to control both LEE expression and motility. The mechanism of RgdR activation of LEE was investigated.

## Results

### T3S screening of EHEC O-island mutants

**Fig. 1:**
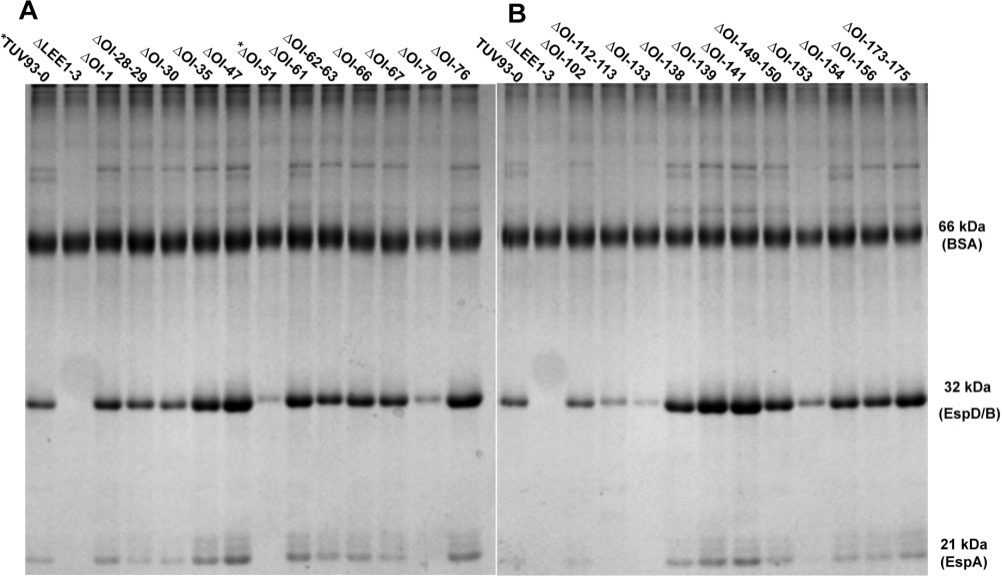
Screening of EHEC O157:H7 O-island (OI) mutants for altered levels of T3S. SDS-PAGE gels showing culture supernatant secretion profiles for EHEC strain TUV93-0 and a selection of 24 isogenic deletion strains. Parent strain TUV93-0 also acted a positive control for T3S and a T3S system mutant (ΔLEE1–3) provided a negative control for secretion. OI deletions are as defined with reference to the original designations in Perna *et al*. (2001). The translocon protein bands are indicated, EspB/D and EspA as well as BSA which was added as a loading control to rule out the precipitation process as a source of variation and to act as a co-precipitant. Strains were cultured in MEM-HEPES to an OD_600_ of 1.0 and culture supernatants were TCA-precipitated, separated by SDS-PAGE and stained with Colloidal blue as described in *Experimental procedures*.

Initial screening identified a subset of OIs with the capacity to either repress or activate T3S in EHEC strains EDL933 and TUV93-0 (Shiga toxin-negative derivative strain of EDL933). For example, TUV93-0 derived mutant's ΔOI-47, ΔOI-76 (Fig. 1A) and ΔOI-141 (Fig. 1B) all had levels of T3S above that of the wild-type parent, suggesting repression by these islands, while ΔOI-51 (Fig. 1A) and ΔOI-133 (Fig. 1B) mutants had reduced levels of T3S, suggesting activation by these islands. In the present study, we focused on the potential significance of OI-51 for colonization and how it controls T3S as variation in this region has been reported to impact on LEE regulation (Yang *et al*., 2009).

### Deletion of OI-51 leads to reduced colonization and shedding levels of *E. coli* O157 strain TUV93-0 *in vivo*

**Fig. 2:**
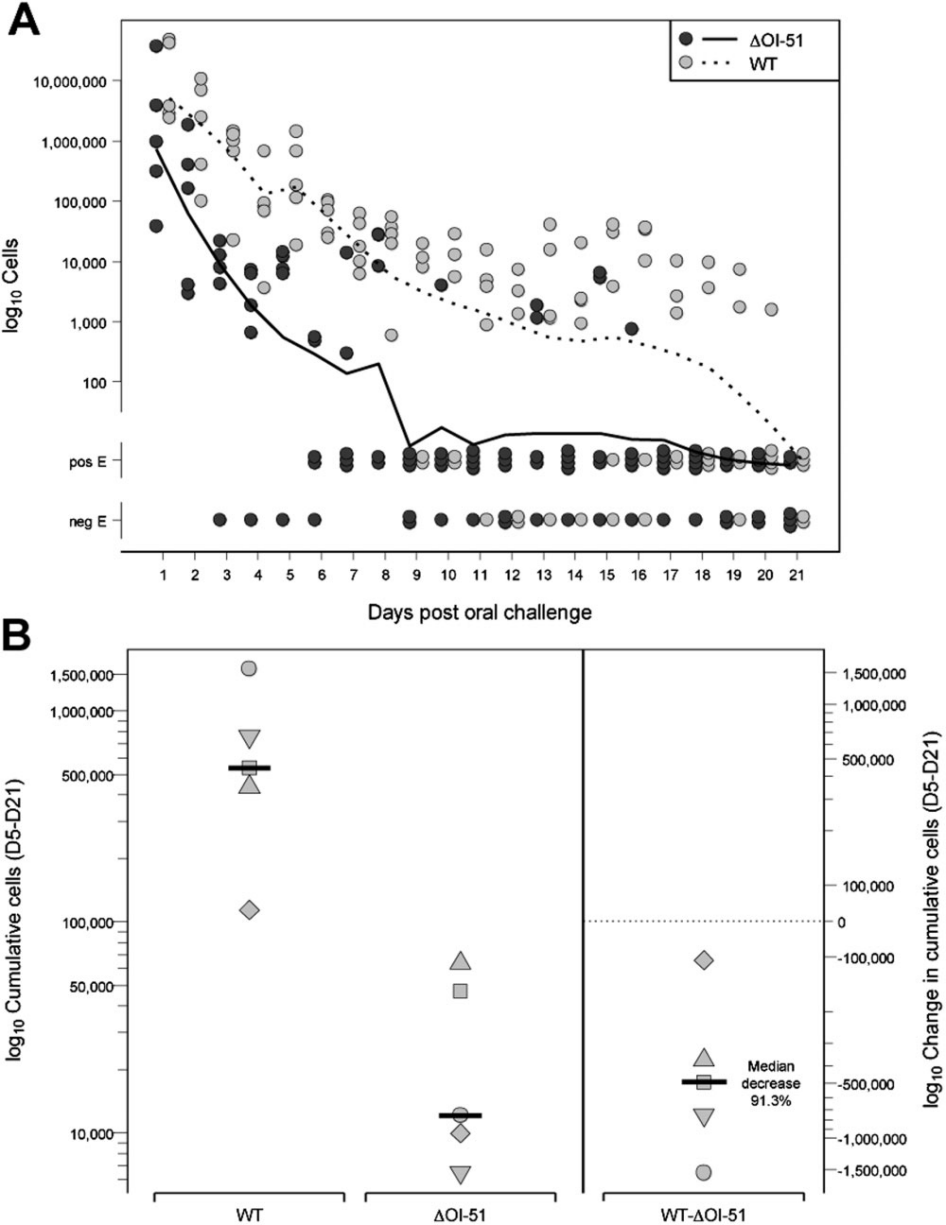
OI-51 contributes to ruminant colonization. Six animals were orally dosed with both wild-type (WT) (TUV93-0) and ΔOI-51 EHEC O157:H7 strains as described in *Experimental procedures*. A. Levels of both strains were determined daily from faecal samples. ‘PosE’ are samples that were positive for the strain following broth enrichment; ‘NegE’ samples were negative following broth enrichment. B. The cumulative shedding levels for each animal and strain were estimated for colonized animals from day 5 onwards to avoid inoculum effects. One animal was excluded from the analysis that was not properly colonized by either strain (< 20 cfu per gram of faeces). The different symbols represent individual animals and allow direct comparison of the two strains in each animal. The right-hand panel of the graph shows the percentage mean decrease of the mutant strain by comparison with the WT. The difference in cumulative shedding levels in the five colonized animals was assessed by a one-sample *t*-test compared to a null hypothesis of 0 change. The data were log10 transformed data to normalize the values, and the difference was statistically significant (*P* = 0.006).

In order to assess the importance of OI-51 for colonization of ruminants, the OI-51 deletion and the parent strain were co-administered in an established ovine colonization model (Wales *et al*., 2001). The OI-51 deletion was marked with a chloramphenicol resistance cassette and the parent strain for resistance to nalidixic acid allowing direct plating and enumeration of the two strains from faeces. Six animals were orally dosed with the two strains, with only one animal failing to be properly colonized (< 20 cfu g^−1^ faeces from the daily counts) (Fig. 2A). For the remaining five animals the estimated relative total levels of the mutant excreted from day 5 onwards, to remove inoculum effects, were estimated with a statistically significant reduction demonstrated for the mutant (*P* = 0.006, Fig. 2B) indicating that OI-51 is important for colonization and persistence in the ruminant GI tract.

### OI-51 sequence analysis

**Fig. 3:**
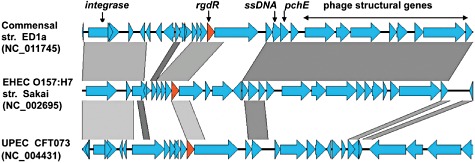
Organization of OI-51 from *E. coli* O157 and nucleotide sequence homology with related regions from *E. coli* ED1a and *E. coli* CFT073. The *E. coli* O157 sequence used for the representation and analysis was from the Sakai strain and the region shown is between the chromosomal co-ordinates: 1594585–1610169 which lie between *ycdF* and *phoQ* of *E. coli* K-12 MG1655. The partially homologous region from *E. coli* CFT073 (chromosomal co-ordinates: 1377764–1393349) lies in the same chromosomal location. The related prophage in *E. coli* ED1a lies between *ydgD* and *ydgG* (chromosomal co-ordinates: 1740689–1756274). The selected regions were compared by blastn; regions and level of homology are indicated by the grey shading with genes encoding RdgR (ECs1581 in Sakai) and homologues (ECED1_1787 in ED1a and C1493 in CFT073) shown in orange. Other characterized genes are annotated as shown with ssDNA indicating a conserved gene in the three regions that is predicted to encode a single-stranded DNA binding protein.

Initial phenotypic screening indicated that an EHEC O157:H7 ΔOI-51 mutant has reduced levels of T3S. OI-51 is a 14.93 kb cryptic prophage designated as CP-933C in EHEC strain EDL933 and Sp7 in EHEC strain Sakai (Hayashi *et al*., 2001) (Fig. 3). The composition of this cryptic prophage varies in other referenced *E. coli* genomes, including *E. coli* CFT073 and ED1a (Fig. 3). Analysis of OI-51/Sp7 genomic structure shows it to be an unusual and highly degraded prophage comprised mainly of P4 phage remnants. The majority of the open reading frames annotated in OI-51/Sp7 are hypothetical although several share features with known proteins, including a P4 integrase (similar to CP4-like integrase and integrase used for 933L and LEE PAI); a P4-like excisionase (Xis); a replication gene similar to the P4 α gene; a putative DNA binding protein similar to P4 ORF88 (AlpA); a putative single-stranded DNA binding protein (ssDNA); a putative transcriptional activator similar to PerC (PchE); and phage structural genes.

### ECs1581 is a positive regulator of T3S in EHEC O157:H7

**Fig. 4:**
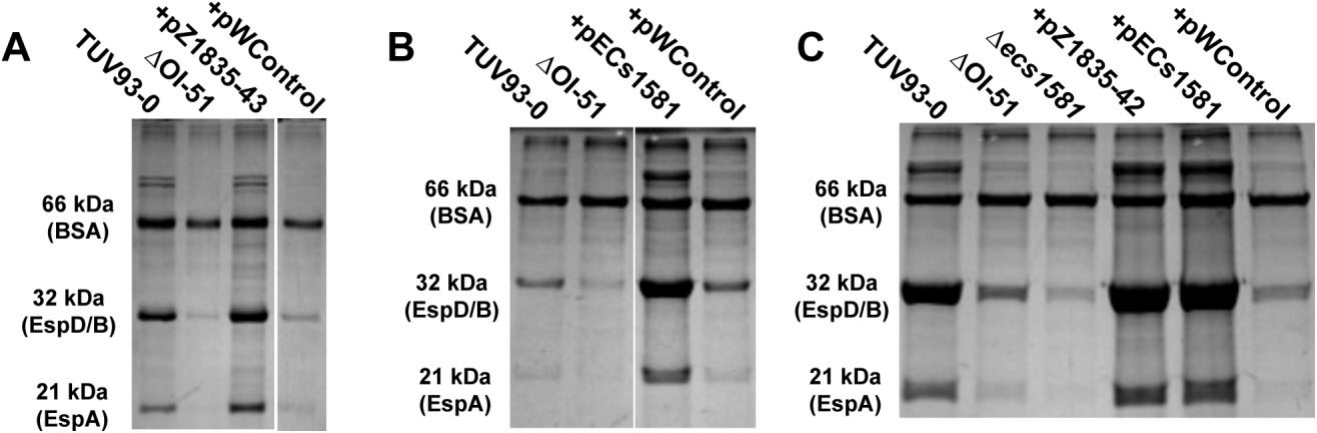
Identification of *ecs1581* as a positive regulator of T3S. A. SDS-PAGE gel showing complementation of T3S in an EHEC ΔOI-51 strain expressing a cloned OI-51 fragment containing genes *z1835-z1843* (pZ1835-pZ1843). The empty vector alone acted as a control (pWControl). B. SDS-PAGE gel showing the T3S profiles for the parent strain TUV93-0 and ΔOI-51 mutant expressing pECs1581 or pWControl. C. SDS-PAGE gel showing the T3S profiles for parent strain TUV93-0, ΔOI-51 and a Δ*ecs1581* mutant expressing pZ1835–Z1843, pECs1581 or pWControl. Protein samples were prepared and analysed as described in *Experimental procedures*.

Systematic analysis of cloned OI-51 regions demonstrated that a 5 kb region (*z1835*–*z1843*) was able to rescue T3S in the TUV93-0 ΔOI-51 mutant (Fig. 4A) when expressed from low copy number vector pWSK29 (pZ1835–43) (Table S1). Deletions across this fragment indicated that a region between *z1841* and *z1842* was required for this activation (data not shown). From the Sakai annotation (Fig. 3), this region does contain an open reading frame, *ecs1581*, the identical sequence being present but not annotated in the EDL933 sequence. Deletion of *ecs1581* by allelic replacement with a resistance cassette significantly reduced T3S levels in a TUV93-0 background. Further, the reduced levels of T3S in both the *Δecs1581* and ΔOI-51 mutants could be complemented by *in trans* expression of *ecs1581* from a low copy number plasmid (pECs1581) (Fig. 4B and C). In line with the animal colonization data, deletion of OI-51 reduced adherence of *E. coli* O157 to bovine epithelial cells and could be complemented by *ecs1581* expressed *in trans* (Fig. S1). We have therefore been able to identify *ecs1581* as a new regulator in EHEC that is able to stimulate T3S and adherence.

ECs1581 is predicted to be a small protein of 99 amino acids with a molecular mass of 11.704 kDa and a pI of 10.09. ECs1581 has no significant sequence similarity with any known protein although it does have a very short section of homology with the spacer region of Ler that bridges the known protein–protein interaction domain of Ler with its DNA binding domain (Mellies *et al*., 2008). However, there is no significant homology of ECs1581 with these functional regions of Ler (Fig. S2).

### Distribution of ECs1581 in *E. coli* strains

**Fig. 5:**
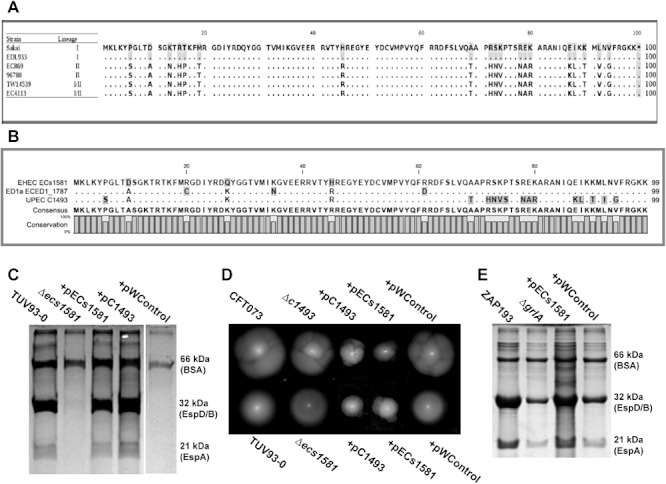
Sequence comparison of ECs1581 variants in *E. coli*. A. Multiple alignment of the predicted amino acid sequences of ECs1581 in different lineages of O157:H7. Residue divergence specific to lineage I EHEC O157:H7 isolates is marked by a pink shaded box. B. Multiple alignment of EHEC variant ECs1581 (EDL933/Sakai) with commensal variant ECED1_1787 (ED1a) and UPEC variant C1493 (CFT073). The RGD motif is at amino acid position 20–22. Residue divergence between the strains is indicated. C. SDS-PAGE gel showing stimulation of T3S in an EHEC Δ*ecs1581* mutant expressing EHEC ECs1581 (pECs1581) or UPEC C1493 (pC1493) from a low copy number plasmid. pWControl alone in the *ecs1581* mutant was used as a negative control strain. TCA-precipitated culture supernatants were analysed as described previously in *Experimental procedures*. D. Analysis of motility repression in an EHEC Δ*ecs1581* and UPEC Δ*c1493* deletion background expressing pECs1581, pC1493 or pWControl. Motility was assessed as described in *Experimental procedures*. E. SDS-PAGE gel showing T3S profile for EHEC strain ZAP193 and isogenic Δ*grlA*::Tn mutant expressing pECs1581 or pWControl. Strains were cultured in DMEM to an OD_600_ of 1.0 and culture supernatant proteins were analysed exactly as described for strains grown in MEM-HEPES.

Sequence analysis of ECs1581 variants in different lineages of EHEC O157:H7 showed the core region of this protein (amino acids 20–68) to be highly conserved; with distinct lineage-specific amino acid substitutions at the N- and C-termini (Fig. 5A). Interestingly, this sequence divergence was specific to LI EHEC O157 by comparison with LII and LI/II (Fig. 5A). Similar analyses of sequences immediately upstream of *ecs1581* showed divergence between these lineages (data not shown) which could potentially impact *ecs1581* expression levels. Orthologues of ECs1581 were also found in sequenced non-O157 EHEC strains (O103:H2, O26:H11 and O111:H-); in EPEC strain O55:H7; newborn meningitis *E. coli* (NMEC) strain IHE3034; enteroaggregative *E. coli* (EAEC) strain O42; atypical EPEC strain E110019; uropathogenic *E. coli* (UPEC) strains CFT073 (O6:K2:H1) and IAI39 (O7:K1); Shigella *spp* and commensal *E. coli* strain ED1a (O81); all with > 77% homology over a region of 99 amino acids (i.e. full-length ECs1581) (Table S2). Multiple divergent copies of *ecs1581* were also present in the chromosomes of strains harbouring this regulator and these are also detailed in Table S2. ECs1581 contains a tri-peptide arginine-glycine-aspartate (RGD) motif (amino acids 20–22, Fig. 5B) which may be important for mediating protein–protein interactions based on previously established functions of this motif (D'Souza *et al*., 1991).

### ECs1581 and orthologous protein C1493 (UPEC) can activate T3S and repress motility in EHEC and UPEC

Given the diversity of *E. coli* strains that harbour sequence-related variants of ECs1581, its prevalence and divergence among different EHEC lineages, and the fact that not all strains with this regulator harbour a LEE-encoded T3S system, we wanted to investigate the functionality of a subset of these protein variants by testing their effects on T3S and the related but more ubiquitous flagella system.

The UPEC CFT073 variant C1493 has 83% sequence similarity to ECs1581 (Fig. 5B) and is also encoded on a cryptic prophage (CP073-3) that is inserted in an identical location on the chromosome as CP-933C (OI-51/Sp7) (Mobley *et al*., 1990; Perna *et al*., 2001; Welch *et al*., 2002). *c1493* was cloned into the low copy number plasmid pWSK29 to create expression plasmid pC1493 and this was assessed for its effects on T3S levels following induced expression in an EHEC *ecs1581* mutant. Expression of pC1493 rescued T3S demonstrating that the variants are functionally interchangeable, despite UPEC lineages not harbouring a LEE-encoded T3S system (Fig. 5C).

To investigate motility, a *c1493* mutant was constructed in UPEC strain CFT073 and examined in soft agar following *in trans* expression of *c1493* and *ecs1581*; in the reciprocal manner motility was examined in a EHEC *ecs1581* mutant strain with *in trans* expression of the two variants. Although only subtle differences in motility were observed for wild-type and mutant constructs in both EHEC and UPEC (Fig. 5D), the *in trans* and presumably higher level expression of the two variants downregulated motility, a phenotype that was not observed for the control strains expressing the empty vector (pWControl) (Fig. 5D).

In EHEC, GrlA is an established regulatory protein encoded by the LEE that positively regulates *ler* transcription and LEE gene expression; and negatively regulates motility in a feedback loop with the repressor GrlR (Iyoda *et al*., 2006). Although unlikely given our observations in EHEC and UPEC, we wanted to rule out the possibility that in EHEC, *ecs1581* may be acting reciprocally on T3S and flagella via *grlA*. We therefore analysed T3S and motility in a *grlA*::Tn mutant (Table S1) with and without induced expression of *ecs1581* from a low copy number plasmid. As expected, T3S levels were reduced in the *grlA*::Tn mutant compared with the parent strain (Fig. 5E). Induced expression of *ecs1581*, however, was able to increase T3S levels in the *grlA*::Tn mutant (Fig. 5E), indicating that *ecs1581* activation of T3S is mediated independently of *grlA*. Further, induced expression of *ecs1581* in the *grlA*::Tn mutant downregulated motility in this strain (data not shown). Other regulators of both T3S and motility include QseB-C (Hughes *et al*., 2009; Kostakioti *et al*., 2009), although RgdR was able to significantly increase T3S levels in a TUV93-0 Δ*qseC* mutant (data not shown) indicating it does not act via this pathway.

### Commensal orthologue ECED1_1787 is unable to stimulate T3S or repress motility in EHEC

**Fig. 6:**
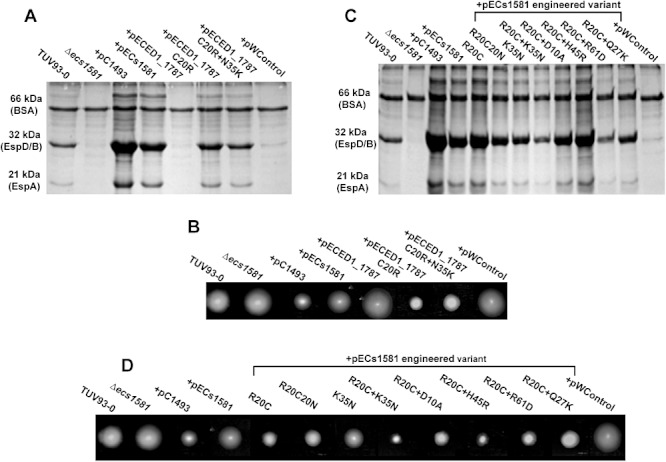
Effects of natural and engineered variants of ECs1581 on T3S and motility. A. SDS-PAGE gel showing T3S levels in EHEC strain TUV93-0 and Δ*ecs1581* mutant expressing wild-type and engineered variants of commensal protein ECED1_1787 from a low copy number plasmid (pECED1_1787, pECED1_1787C20R and pECED1_1787N35K). pWControl in the *ecs1581* mutant strain was used as a negative control and wild-type variants pECs1581 and pC1493 were included as a reference for high T3S levels. B. Analysis of motility in strain TUV93-0 and Δ*ecs1581* mutant expressing pECED1_1787, pECED1_1787C20R, pECED1_1787N35K or pWControl. C. SDS-PAGE gel showing T3S levels in strain TUV93-0 and Δ*ecs1581* mutant expressing wild-type and engineered variants of ECs1581 from a low copy number plasmid (pC1493, pECs1581, pECs1581R20C, pECs1581R20C20N, pECs1581K35N, pECs1581R20C + K35N, pECs1581R20C + D10A, pECs1581R20C + H45R, pECs1581R20C + R61D, pECs1581R20C + Q27K and pWControl). D. Analysis of motility in the same strain set. Analysis of culture supernatants and motility was carried out as described in *Experimental procedures*. The motility figures (B and D) are composite images from photographs of motility agar plates inoculated with the strain of interest. The same images are used for the wild types and controls in B and D.

Commensal strain ED1a (a close relative of UPEC strain CFT073; phylogenetic group B2) also harbours an orthologue of ECs1581, ECED1_1787, which has 94% sequence similarity (Fig. 5B) (Clermont *et al*., 2008; Jaureguy *et al*., 2008; Touchon *et al*., 2009). Despite having the highest known homology to ECs1581, even compared to other EHEC O157:H7 variants, commensal protein ECED1_1787 was unable to activate T3S or repress motility following induced expression (pECED1_1787) in an EHEC *ecs1581* mutant strain (Fig. 6A). This was informative as there are only six amino acid differences between ECs1581 and ECED1_1787 (Fig. 5B); and by the finding that C1493 has this capacity despite its lower homology to ECs1581 (83% versus 94% respectively). The residue divergence in ECED1_1787 by comparison to ECs1581 maps to the following residues: D10A, R20C, Q27K, K35N, H45R and R61D (amino acids 1–99) (Fig. 5B). Interestingly, two of these changes occur within predicted domains with a D10A substitution in a putative N-myristylation motif (amino acids 7–12), and an R20C substitution in the EHEC-conserved RGD motif (amino acids 20–22) (Fig. 5B). Like ECED1_1787, C1493 has a D10A but harbours an RGD motif as in ECs1581 (Fig. 5B).

### Amino acid substitutions in ECs1581 and ECED1_1787 alter their regulation of T3S and motility

Based on the functional differences demonstrated for the ECs1581 and ECED1_1787 proteins, a series of reciprocal substitutions in proteins ECs1581 and ECED1_1787 were constructed by site-directed mutagenesis and their effects on T3S and motility assessed in an EHEC *ecs1581* mutant. A single C20R mutation in protein ECED1_1787 (pECED1_1787C20R) was sufficient to enable this variant to now activate T3S and repress motility (Fig. 6A). An ECED1_1787 C20R + N35K double mutation (pECED1_1787C20R + N35K) did not increase T3S levels over the single C20R change (Fig. 6A). Strikingly, induced expression of ECED1_1787C20R severely repressed motility (Fig. 6B). This shift in phenotype between wild-type ECED1_1787 and engineered variant ECED1_1787C20R was intriguing as induced expression of ECED1_1787 in the same strain background, if anything, increased motility. A K35N substitution in ECs1581 (ECED1_1787N35) attenuated its stimulation of T3S in an EHEC *ecs1581* mutant when induced (pECs1581K35N) from a low copy number plasmid (Fig. 6C). Surprisingly, an R20C change in the EHEC-conserved RGD motif (ECED1_1787C20) slightly increased T3S levels following induced expression (pECs1581R20C) in this background, and this engineered variant was also more repressive for motility than wild-type ECs1581 (Fig. 6C and D). A further R20C20N mutation in this same RGD motif, however, did reduce T3S levels compared to wild-type ECs1581 (Fig. 6C). Although the small size of RgdR means that some changes could affect protein conformation and stability, these results highlight the functional importance of residue 20 and other targeted combinations for the regulatory activities of this novel regulator.

### Mechanism of ECs1581 activation of T3S

**Fig. 7:**
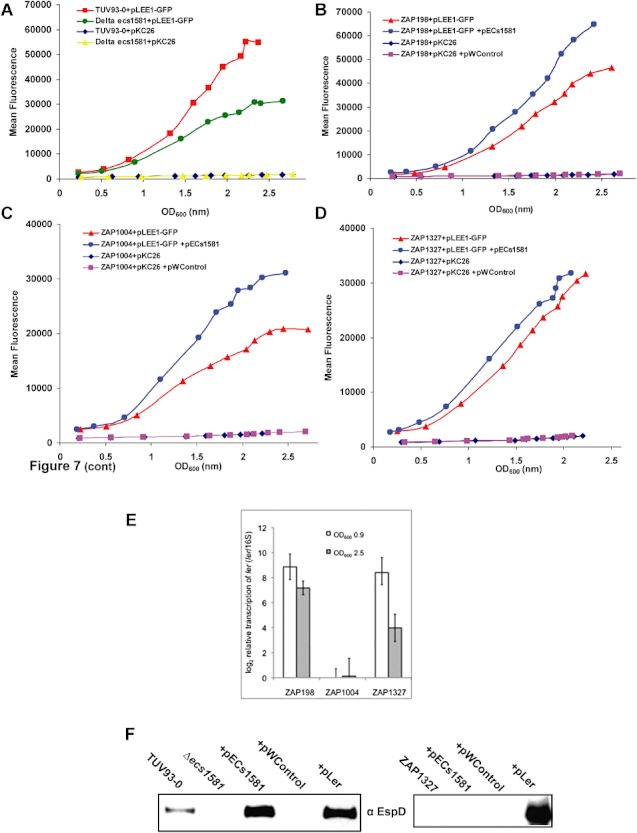
ECs1581 stimulates T3S via LEE1. A. Measurement of LEE1 promoter activity in EHEC strain TUV93-0 and isogenic Δ*ecs1581* mutant. A 428 bp LEE1 promoter fragment (−444 to −16 upstream from the *ler* ATG start codon) was cloned into the promoter-less green fluorescence protein (GFP) plasmid pKC26 to create transcriptional fusion plasmid pLEE1–GFP. Strains transformed with this reporter were cultured in DMEM and the fluorescence produced by each bacterial population measured every 60 min as described in the *Experimental procedures*. Corresponding OD_600_ measurements were taken at each time point and plotted against the mean fluorescence intensity values for each strain. The promoter-less plasmid pKC26 in each strain background acted as a control. B. Measurement of the pLEE1–GFP reporter and control plasmids in EHEC strain ZAP198 (Table S1) with and without induced expression of *ecs1581* (pECs1581) from low copy number plasmid pWSK29. C. Measurement of the pLEE1–GFP reporter and control plasmids in strain ZAP198*Δler* (ZAP1004, Table S1) with and without induced expression of pECs1581. D. Measurement of the pLEE1–GFP reporter and control plasmids in strain ZAP198 with a constitutive LEE1 promoter (ZAP1327, Table S1) with and without induced expression of pECs1581. E. RT-PCR measurement of *ler* expression levels in EHEC strains ZAP198, ZAP1004 and ZAP1327. F. Western blot of EspD secretion by EHEC Δ*ecs1581* and the replaced LEE1 promoter mutant (ZAP1327) *trans* expressing Ler (pLer) or ECs1581 (pECs1581) from low copy number plasmids.

To examine how ECs1581 activates T3S, its capacity to stimulate a plasmid-encoded LEE1–GFP transcriptional fusion was studied in different genetic backgrounds. Population fluorescence levels from the LEE1 fusion were reduced significantly in an *ecs1581* mutant (Fig. 7A). Ler, expressed from LEE1, is required for autoregulation of LEE1 and activation of the remaining LEE operons leading to T3S. We determined whether ECs1581 was able to stimulate LEE1 expression in the absence of *ler* autoregulation. Population fluorescence levels from the LEE1 reporter were determined in EHEC strains ZAP198 (WT, Table S1) and ZAP1004 (Δ*ler*, Table S1) following induction of *ecs1581* (pECs1581) (Fig. 7B and C). ECs1581 was able to stimulate LEE1 expression in the absence of *ler* (Fig. 7C). To determine if ECs1581 regulation of LEE1 expression in EHEC is direct or indirect, electrophoretic mobility shift assays (EMSA) were carried out using a 291 bp LEE1 promoter fragment (−288 to +3) and purified ECs1581. While it was evident that the protein bound to the DNA in a concentration-dependent manner (Fig. S3), ECs1581 also bound to the control DNA fragment, indicating a lack of specificity to this interaction. Of note was the finding that the commensal variant ECED1_1787, which was unable to activate T3S, was unable to bind the same LEE1 promoter fragment indicating that the regulatory activity of ECs1581 may require DNA binding (Fig. S3).

In order to confirm that ECs1581 stimulates T3S through activation of the Ler autoregulatory cascade, a strain (ZAP1327, Table S1) was constructed in which the LEE1 promoter was replaced by a cassette that allowed constitutive expression of *ler*, removing the normal transcriptional regulation of LEE1 including sequences required for *ler* autoregulation (Mellies *et al*., 2008; Yerushalmi *et al*., 2008). Constitutive *ler* transcription in this replaced promoter strain was confirmed by RT-PCR (Fig. 7E). There was, however, no detectable T3S from this strain (Fig. 7F). Induced Ler expression in this background was able to stimulate T3S but this was not the case for induced ECs1581. ECs1581 induction of the LEE1 reporter fusion in this strain background was detectable but remained at a constant level throughout the growth curve (Fig. 7D). This was in stark contrast to the activation kinetics measured for the LEE1 fusion in the strain with an unaltered LEE1 promoter on the chromosome. In this wild-type background, the stimulation of the LEE1 fusion increased exponentially with optical density, whereas this increase was restricted in the strain with a replaced LEE1 chromosomal promoter. Therefore, stimulation of T3S by ECs1581 occurs through initial activation of transcription from the LEE1 promoter, leading to induction of the Ler autoregulatory cascade that in turn promotes expression of the remaining LEE operons and T3S.

## Discussion

The contribution of bacteriophages to intra- and inter-species bacterial evolution and adaptation are well documented. Enterobacteriaceae in particular are exposed to a plethora of phages in the mammalian GI tract and many of these phages carry additional genetic information that allow the host bacteria to adapt to local environments and occasionally cause disease. EHEC is an important attaching and effacing pathogen that can cause serious complications in at-risk individuals due to the production of phage-encoded Shiga toxins. T3S is an essential virulence factor for EHEC which is required for successful colonization of the ruminant host. This virulence secretion system is encoded by the LEE pathogenicity island and prophage-encoded effector proteins are able to utilize this system to be exported directly into host cells to sabotage host communication pathways and promote bacterial colonization.

In this study, a number of prophage deletions (OIs) were examined for their effect on T3S levels in order to identify horizontally acquired regions that cross-talk with this essential colonization system. Of the 24 OI mutants studied in TUV93-0 (excluding the LEE1-3 deletion), five had lower levels of T3S (Fig. 1A and B), including OI-51. OI-51 is a 14.93 kb region in EDL933/TUV93-0 and 15.46 kb in the sequenced Sakai strain. Variations of this island are present in all EHEC O157:H7 strains as well as non-O157 EHEC strains, but also in specific UPEC, NMEC and commensal *E. coli* strains that do not contain a LEE-encoded T3S system. OI-51 in EDL933 contains a predicted 22 open reading frames, with a subset indicating P4 ancestry. Deletion of OI-51 reduced persistence in competitive ruminant colonization experiments indicating the potential importance of this region.

Through a combination of deletion and complementation analyses, *ecs1581* was identified as a gene able to restore T3S levels in both the OI-51 and *ecs1581* mutants. Three lineages of EHEC O157:H7 have been proposed based on genome sequences (Kim *et al*., 1999; Zhang *et al*., 2007). In the present study, two main sequence types of ECs1581 were identified, LI and LII & LI/II, the latter having identical protein-coding sequences. EHEC O157:H7 lineage-specific variants of ECs1581 (LI) from strains TW14539 (LI/II) and 96788 (LII) were cloned and assessed for their effects on T3S and motility. All the variants were able to induce T3S and repress motility, although slight variation in induction levels was observed (Fig. S4A and B). Sequences immediately upstream of these two variants also differ, but it is not known if expression levels of these two variants differ as these were not assessed in this study. Variation in activity and/or expression of ECs1581 could therefore contribute to differences in T3S expression demonstrated between EHEC O157:H7 strains (Son *et al*., 2002; Roe *et al*., 2003; Rashid *et al*., 2006; Yang *et al*., 2009). We also note that genetic variability among the different EHEC O157:H7 lineages are associated with regions adjacent to *pchD* and *pchE* genes on OI-43/48 (the tellurite resistance and adhesin-conferring islands) and OI-51 respectively (Zhang *et al*., 2007 and Yang *et al*., 2009).

Variants of the protein from different *E. coli* strains exhibited markedly different capacities to regulate T3S. A variant present in the commensal strain ED1a did not activate T3S and contains an R20C (CGD) substitution in the EHEC-conserved RGD motif. Replacement of this residue to generate an RGD motif in the commensal variant produced a protein that now had the capacity to activate T3S. Based on this we have termed the EHEC regulator RgdR. It was interesting to note that regardless of the effect on T3S, all variants in RgdR had a marked impact on motility. In general, there was an inverse correlation between T3S and motility that was also apparent for several of the engineered variants. However, there were clear exceptions to this, for example ECs1581 R20C + K35N had a weak capacity to activate T3S yet unusually, showed the strongest capacity to inhibit motility. Additional work confirmed that this regulation of T3S and motility was independent of *grlA*, previously demonstrated to repress motility on induction of T3S (Iyoda *et al*., 2006). RgdR is therefore identified as a novel regulator able to co-ordinate T3 and motility expression. Also of note was the finding that expression of different variants could increase bacterial binding to Congo red (Fig. S4C). Although the mechanism of this Congo red binding was not investigated in this study, recent work by Lee *et al*. (2011) has demonstrated that EDL933 is able to produce curli fibres. Taken together, the pleiotropic phenotypes displayed by RgdR including on T3S, motility and cell surface charge suggests it may play a more global role in the cell. This would explain the presence of variants of this regulatory protein in strains without a LEE-encoded T3S system. Future work will address the regulons of RgdR in EHEC O157:H7 and strains not harbouring a LEE pathogenicity island.

RgdR was able to stimulate LEE1 expression independently of Ler and required a normal LEE1 promoter region for this activation. A construct that resulted in constitutive expression of LEE1 could be induced to secrete EspD by increased expression of Ler *in trans* but not by induced expression of RgdR *in trans*. In this background, RgdR was limited in its capacity to induce a LEE1–GFP reporter fusion construct. An explanation for this is that even though RgdR can activate the LEE1 promoter in the absence of Ler, Ler expression and the Ler auto-induction cycle is required for the activation of T3S as Ler, and not RgdR, activates transcription of LEE2-5 encoding the T3S system and secreted proteins. One explanation for the data is that ECs1581 could inhibit Ler autorepression at the LEE1 promoter. It has been proposed that high Ler levels allow binding of Ler to a low affinity site which may repress transcriptional activation. The kinetics of LEE1 activation in the presence of induced ECs1581 would fit this model. Repeated attempts to purify functional Ler to test possible interactions were unsuccessful. We note that ECs1581 does share homology with Ler over a linker region (Fig. 4D) that could be involved in this interaction.

RgdR did demonstrate a low affinity and non-specific interaction with DNA (Fig. S3), a property that was absent from the ECED_1787 variant that did not stimulate T3S. We propose that RgdR may act as a co-factor with one or more regulators of T3S (Tree *et al*., 2009; Kendall *et al*. 2010) to control transcription from the LEE1 promoter. One regulatory network investigated was the quorum-sensing two-component system, QseBC, as this controls both T3S and motility (Hughes *et al*., 2009; Kostakioti *et al*., 2009). However, RgdR was able to significantly increase T3S levels in a TUV93-0 Δ*qseC* mutant (data not shown). Another possibility is that RgdR works with the Pch family of LEE activators (Iyoda and Watanabe, 2004; Porter *et al*., 2005; Abe *et al*. 2008; Yang *et al*., 2009), although there is no significant homology between the two, in particular to the region truncated in the smaller Pch variants (PchC–E) (Abe *et al*. 2008). With the exception of *pchD*, all *pch* alleles are encoded on prophage elements, and it is hypothesized that multiple prophages in the chromosome of EHEC O157:H7 may be able to recombine to affect the total copy number of *pch* genes; thus potentially impacting on levels of LEE gene expression (Ohnishi *et al*., 2001; Iyoda and Watanabe, 2004; Asadulghani *et al*., 2009). As with the Pch activators of LEE, all members of the RgdR family of proteins are prophage-associated. For example, EHEC strain EDL933 harbours two RgdR truncates (both 63 amino acids), *z1197* and *z1636* that are located on OI-43 and OI-48 respectively (Table S2). In EHEC strain Sakai only one truncate exists (63 amino acids), *ecs5415*, which is located on Sakai prophage-like element 1 (SpLE1). In EHEC O26:H11 strain 11368, four copies of RgdR can be found (Table S2); three full-length (99 amino acids) variants and one truncate (63 amino acids) which is located on OI-48. Perhaps, as proposed for the Pch proteins, genome integration of CP-933C (OI-51) and similar prophages may additively influence levels of LEE gene expression in EHEC. It is therefore perhaps not surprising that a number of deleted prophage regions analysed in this study and previously (Tree *et al*., 2011) have a regulatory impact on T3S. The RgdR, Pch and Psr families of regulators provide clear examples of how differences in the prophage repertoire of EHEC strains can impact on expression of critical colonization factors and are a major driving force in the evolution of this pathogen (Asadulghani *et al*., 2009; Tree *et al*., 2009; 2011).

In summary, this research has defined an important region (OI-51) necessary for EHEC O157:H7 colonization and persistence in ruminants, and has identified a completely new family of small bacterial regulators that control gene expression in *E. coli*.

## Experimental procedures

### Bacterial strains, plasmids and primers

All bacterial strains, plasmids and primers used in this study are detailed in Table S1.

### Bacterial culture conditions and media

Bacterial strains were routinely cultured at 37°C with shaking in Luria–Bertani (LB) broth or agar. In the analysis of T3S proteins, either DMEM (Sigma-Aldrich) or MEM-HEPES (Sigma-Aldrich) supplemented with 250 nM Fe(NO_3_)_2_ and 0.2% glucose was used for culturing strains. When measuring promoter activity with GFP transcriptional fusions, strains were cultured in DMEM. In Congo red binding assays, CFA agar (LabM) and CFA broth [0.15% tryptone yeast extract (Becton Dickinson); 1% casamino acids (Oxoid); 0.01% Congo red (Sigma-Aldrich); 400 µM MgSO_4_.7H_2_O and 40 µM MnCl_2_.4H_2_O] was used for selecting and culturing colonies respectively. When required, Isopropyl β-D-1-thiogalactopyranoside (IPTG), 5-bromo-4-chloro-3-indolyl-beta-D-galactopyranoside (X-gal) and antibiotics were added to media at the following final concentrations: 1 mM IPTG, 20 ng ml^−1^ X-gal, 50 µg ml^−1^ ampicillin, 50 µg ml^−1^ chloramphenicol, 100 µg ml^−1^ kanamycin and 15 µg ml^−1^ nalidixic acid.

### OI-51 ruminant colonization study

Crossbred lambs (six 6-week-old) were housed in bio-secure containment level 2 accommodation and supplied with food and water *ad libitum*. All lambs were confirmed to be free of EHEC O157 by enrichment and O157 immuno-magnetic separation prior to commencing the studies. Lambs were dosed orally with 1 × 10^10^ cfu of wild-type *E. coli* O157:H7 and mutant bacteria (5 × 10^9^ cfu of each). Inocula, 10 ml resuspended in 10 ml of PBS pH 7.4, were delivered using a worming gun (Novartis Animal Health) ensuring that the whole inoculum was delivered directly to the pharynx. Rectal faecal samples from each lamb were collected daily for direct plating onto sorbitol-MacConkey (Oxoid) plates supplemented with appropriate antibiotics. Enrichment was carried out on samples in buffered peptone water for 6 h at 37°C and then plated onto sorbitol-MacConkey plates supplemented with the appropriate antibiotic. Representative colonies were confirmed to be *E. coli* O157 by latex agglutination (Oxoid). Animal experiments were performed in line with the Animals Scientific Procedures Act (1986) and were approved by the local ethical review committee.

### Construction of mutations in EHEC and UPEC

Defined *ecs1581*, *c1493* and *qseC* mutants were constructed using allelic exchange as described previously by Blomfield *et al*. (1991) and Emmerson *et al*. (2006). Briefly, chromosomal regions (∼ 600–1000 bp) flanking the genes of interest were PCR-amplified (Table S1) with a high fidelity Phusion polymerase (Fiinzyme) and cloned into a chloramphenicol-resistant temperature-sensitive exchange plasmid (pIB073) (Table S1). A kanamycin resistance cassette (Gally *et al*., 1994) was then cloned in between the homologous flanking regions on the exchange plasmid and subjected to several rounds of temperature and antibiotic selection to achieve allelic replacement in the relevant strain background. All mutants were verified by PCR and successful complementation was achieved by providing the related genes *in trans* on low copy number plasmid pWSK29 (Table S1).

### Construction of plasmids for expression and complementation

Complementation plasmids were constructed using PCR products amplified with high fidelity Phusion polymerase cloned into pWSK29 (Table S1). *E. coli* strain DH5α was used as the intermediate host strain for cloning and all constructs were verified by sequencing.

### Construction of engineered ECs1581 and ECED1_1787 protein variants

Site-directed mutagenesis was carried out using an overlap PCR approach with the primers listed in Table S1. Briefly, the 5′ and 3′ regions of each gene were PCR-amplified with engineered mutations in the relevant primers. The two PCR products were then used as a template for the overlap PCR using the outside primers. The PCR products were cloned into vector pWSK29, verified by sequencing (Table S1), and their effects on motility and T3S in a TUV93-0 Δ*ec1581* mutant background assessed.

### Measurement of LEE1 promoter activity

In order to assess LEE1 gene expression in both wild-type and mutant *E. coli* O157:H7 backgrounds, a 428 bp PCR-generated DNA promoter fragment (−444 to −16 bp upstream from the *ler* ATG start codon) was amplified from strain TUV93-0 using high fidelity Phusion polymerase, and cloned into the promoter-less green fluorescence protein (GFP) plasmid pKC26 to create transcriptional fusion plasmid pLEE1–GFP (Table S1). Test strains harbouring this reporter were cultured in DMEM supplemented with chloramphenicol and the GFP produced by each bacterial population was measured every 60 min by transferring 200 µl aliquots of culture into triplicate wells in a black 96-well plate (FluoroNunc) and reading the plate in a fluorimeter (FLUOstar Optima) using 485 nm absorbance and 520 nm emissions. Promoter-less plasmid pKC26 in each strain background acted as a control for auto-fluorescence. When LEE1 promoter activity was measured in response to induced expression of *ecs1581* from low copy number plasmid pWSK29 (pECs1581), strains were cultured in DMEM supplemented with chloramphenicol, ampicillin and IPTG (1 mM). All were tested in triplicate and on at least three separate occasions.

### Congo red binding assay

Strains were initially streaked onto CFA agar plates (LabM) before individual colonies were taken and inoculated into CFA broth and grown overnight at 37°C with shaking. Cultures were diluted in PBS to an OD_600_ of 1.0, centrifuged at 4000 *g* for 5 min and resuspended in 1 ml of sterile PBS. Washing was repeated a further two times to remove all traces of culture broth before finally resuspending the culture pellets in 2.5 ml of PBS containing a final concentration of 0.01% Congo red. Samples were incubated at 37°C for 30 min before a 1 ml aliquot was taken and centrifuged at 10 000 *g* for 10 min. Supernatants were discarded and pellets were air-dried and photographed using a Lumina digital camera. All strains were tested in triplicate and on three separate occasions.

### Motility assays

Motility plates were prepared by adding 0.3% agar (Sigma) to 1% Bacto-Tryptone (BD; Becton Dickinson) broth containing 0.5% NaCl (Sigma-Aldrich). For plasmid-based complementation experiments, IPTG and ampicillin was added to plates when appropriate and all plates were poured the night before use and allowed to air-dry on the bench. Plates were stab inoculated and incubated at 37°C for 16 h. All strains were tested in triplicate and each experiment was carried out on three separate occasions.

### Adherence assays

Bacteria for the assays were cultured in MEM-HEPES and added at an moi of approximately 100 bacteria to each epithelial cell. Embryonic bovine lung cells (no.21 ACC192, German Collection of Microorganisms and Cell Cultures) were cultured in MEM-HEPES in eight-well chamber slides. The cells were washed with PBS and incubated in unsupplemented MEM-HEPES for 1 h before they were infected with bacteria. After 180 min, bacteria and EBL cells were fixed by incubating in 4% paraformaldehyde for 30 min and then washed three times with PBS. The samples were incubated for 90 min with anti-O157 LPS antibody (MAST Group) at 1:4000 and following washes incubated with Alexa Fluor 594-conjugated goat anti-rabbit immunoglobulins antibody (1/1000, Molecular Probes) for 1 h. The slides were washed and mounted (Hydromount, National Diagnostic). Imaging was carried out using a Leica fluorescence microscope and Open Lab software (PerkinElmer).

### Preparation and analysis of T3S culture supernatant proteins

Bacterial strains were cultured overnight in LB broth at 37°C with shaking before being diluted to a starting optical density (OD_600_) of 0.05 in MEM-HEPES (supplemented with glucose and iron). Cultures were grown to a final OD_600_ of 1.0 then centrifuged at 4000 *g* for 30 min at 4°C and supernatants filtered through 0.45 µm low protein-binding filters (Millipore). A 10% (v/v) final concentration of trichloroacetic acid (TCA; Sigma-Aldrich) was used to precipitate the proteins and BSA (NEB) (4 µg ml^−1^) was added to act as a co-precipitant. Supernatants were incubated overnight at 4°C and centrifuged at 4000 *g* for 30 min at 4°C. Protein pellets were air-dried and resuspended in an appropriate volume of resuspension buffer (1.5 M Tris-HCL) in order to standardize samples and to take into account the slight variation in OD_600_ at which cultures were harvested. Culture supernatant proteins were subsequently analysed through a 12% SDS-PAGE and visualized by Colloidal blue staining (Severn Biotech). Gel images were captured using a Flowgen MultiImage light cabinet and ChemiImager 4000i v.4.04 software.

### Protein detection

SDS-PAGE separated proteins were transferred onto Hybond ECL nitrocellulose membrane (Amersham Biosciences) using a Trans-Blot electrophoretic transfer cell (Bio-Rad). Nitrocellulose membranes were blocked with 8% (w/v) dried milk powder (Marvel) in PBS at 4°C overnight and incubated with the relevant antibodies diluted in wash buffer [1% dried milk (Marvel) and 0.05% (v/v) polyoxyethylenesorbitan monolaurate (Tween 20, Sigma-Aldrich) in PBS] at the following dilutions: mouse monoclonal anti-EspD (kindly provided by Professor T. Chakraborty, University of Giessen) and rabbit polyclonal anti-mouse IgG HRP conjugated antibodies (Dako) were both diluted 1:4000. All antibody-membrane incubations were carried out for 1 h at RT on a platform shaker and were washed for 3 × 10 min in wash buffer before and after each antibody step. For ECL detection, membranes were incubated in 2.5 ml of ECL Solution 1 (Amersham) mixed with 2.5 ml of ECL Solution 2 (Amersham) for 5 min at RT on the bench. Chemiluminescence was detected on Hyperfilm ECL chemiluminescence film (Amersham) developed in a Protec automatic film processor (Optimax). Images were taken using a Flowgen MultiImage light cabinet and ChemiImager 4000i v.4.04 software.

### Native protein purification

*Escherichia coli* BL21 (DE3) (Invitrogen) cells harbouring plasmid pET28a-ECs1581, pET28a-ECED1_1787 or pET28a-ECED1_1787C20R were grown in LB broth in the presence of kanamycin at 37°C with shaking to an OD_600_ of 0.5. Cultures were induced with IPTG and grown for a further 4 h under the same conditions before harvesting by centrifugation at 4000 *g* for 30 min. Cell pellets were suspended in lysis buffer (50 mM NaH_2_PO_4_, 300 mM NaCl, 10 mM imidazole and 1 mg ml^−1^ lysozyme, pH 8.0) and lysed by sonication (6 × 10 s pulses with a 10 s cooling period on ice in between each pulse). Lysates were centrifuged at 10 000 *g* at 4°C for 30 min, incubated with Ni2^+^ NT resin (Qiagen) for 1 h at RT on a platform shaker and loaded onto a purification column (Bio-Rad). The column was washed twice with wash buffer (50 mM NaH_2_PO_4_, 300 mM NaCl, 20 mM imidazole, pH 8.0) and the protein was eluted from the column in elution buffer (50 mM NaH_2_PO_4_, 300 mM NaCl, 250 mM imidazole, pH 8.0). Crude preps were transferred into dialysis tubing (8000 kDa molecular weight cut-off, Medicell international) and dialysed in 2 l of sterile PBS which was replenished every 24 h for a total of three changes. Total protein concentration was determined by BCA according to the manufacturer's instructions (Novagen) and purity was verified by SDS-PAGE and antibody detection of recombinant His-tagged proteins. Purified proteins were subsequently used in electrophoretic mobility shift and *in vitro* transcription assays along with PCR generated DNA promoter fragments.

### Electrophoretic mobility shift assay

To determine if ECs1581 activation of LEE1 is direct or indirect, a 291 bp LEE1 promoter (−288 to +3, with respect to the *ler*ATG start codon) and a control *gapA* fragment (also 291 bp) were PCR-amplified (Table S1) from EHEC strain TUV93-0 and subjected to EMSA with either purified ECs1581, ECED1_1787 or ECED1_1787C20R proteins. EMSA was carried out as described previously (Tree *et al*., 2011). Briefly, the purified proteins were co-incubated with concentrations of ddUTP-11-DIG (Roche) end-labelled DNA in binding buffer [10 mM Tris (pH 8.0), 50 mM KCl, 5 mM EDTA, 200 µg ml^−1^ BSA, 100 ng ml^−1^ poly d(I-C), 50 mM glutamate and 5% glycerol] for 30 min at 25°C and loaded onto 5% non-denaturing polyacrylamide gels in 0.5X TBE. Fifty nanograms of unlabelled DNA was used for competition experiments and added to the binding reaction. The DNA and DNA–protein complexes were electro-transferred onto nylon membranes and developed with AP-conjugated anti-DIG antibody (Roche) as per the manufacturer's instructions.

### RT-qPCR

Total RNA was purified from the bacteria using an RNeasy kit (Qiagen) and random primers used to reverse transcribe the RNA using Affinityscript (Stratagene). qPCR was carried out with a PowerSybr mastermix (Applied Biosystems) and amplified in a MxPro 3000 qPCR machine (Stratagene). The qPCR primers used are listed in Table S1. Transcript abundance was normalized to 16SrRNA and relative transcription calculated using MxPro software (Stratagene).

### Bioinformatic analyses

Variants of ECs1581 were identified using blastp (http://www.ncbi.nlm.nih.gov) and sequences were obtained from GeneBank (http://www.ncbi.nlm.nih.gov/GenBank). Putative hypothetical domains in ECs1581 were identified using MyHits (http://myhits.isb-sib.ch/cgi-bin/index) and KEGG (http://www.genome.jp/kegg). OI-51 prophage alignments were generated using Easyfig (http://easyfig.sourceforge.net/). Sequence alignments were performed using ClustalW2 (http://www.ebi.ac.uk/Tools/msa/clustalw2), EMBOSS Align (http://www.ebi.ac.uk/Tools/emboss/align) and LALIGN (http://www.ch.embnet.org/software/LALIGN_form.html). CLC Sequence Viewer (http://www.clcbio.com) was used to construct and visualize alignments among the protein-coding regions of ECs1581; the ECs1581 region was identified in un-annotated draft *E. coli* sequences through blastn searches.

### Sequencing

All DNA sequencing was carried out by GenePool, School of Biological Sciences, University of Edinburgh, and was performed using an ABI Prism BigDye terminator cycle sequencing kit version 3.1 (Applied Biosystems). Reactions were analysed on an ABI 3730 DNA sequencer.
