# Supplementary material for: Identification of a novel prophage regulator in Escherichia coli controlling the expression of type III secretion
Source: Mol Microbiol. 2011 Dec 9;83(1):208–23. doi: 10.1111/j.1365-2958.2011.07927.x (PMC3378721; doi:10.1111/j.1365-2958.2011.07927.x)
Supplement: Supplementary file 1 [file mmi0083-0208-SD1.pdf]

Table S1. List of strains, plasmids and primers used in this study

Strains/plasmids/primers	Description	Source/reference
<u>**Strains**</u>		
TUV93-0	Shiga toxin-negative derivative of EHEC O157:H7 strain EDL933	Campellone *et al.*, 2004
TUV93-0 ΔLEE1-3 (Cam^R)	Lambda red generated LEE1-3 (OI-148A) mutant in strain TUV93-0	John Leong, (TUFTS)
TUV93-0 ΔOI-1 (Cam^R)	Lambda red generated OI-1 mutant in strain TUV93-0	John Leong
TUV93-0 ΔOI-28-29 (Cam^R)	Lambda red generated OI-28-29 mutant in strain TUV93-0	John Leong
TUV93-0 ΔOI-30 (Cam^R)	Lambda red generated OI-30 mutant in strain TUV93-0	John Leong
TUV93-0 ΔOI-35 (Cam^R)	Lambda red generated OI-35 mutant in strain TUV93-0	John Leong
TUV93-0 ΔOI-47 (Cam^R)	Lambda red generated OI-47 mutant in strain TUV93-0	John Leong
TUV93-0 ΔOI-51 (Cam^R)	Lambda red generated OI-51 mutant in strain TUV93-0	John Leong
TUV93-0 ΔOI-61 (Cam^R)	Lambda red generated OI-61 mutant in strain TUV93-0	John Leong
TUV93-0 ΔOI-62-63 (Cam^R)	Lambda red generated OI-62-63 mutant in strain TUV93-0	John Leong
TUV93-0 ΔOI-66 (Cam^R)	Lambda red generated OI-66 mutant in strain TUV93-0	John Leong
TUV93-0 ΔOI-67 (Cam^R)	Lambda red generated OI-67 mutant in strain TUV93-0	John Leong
TUV93-0 ΔOI-70 (Cam^R)	Lambda red generated OI-70 mutant in strain TUV93-0	John Leong
TUV93-0 ΔOI-76 (Cam^R)	Lambda red generated OI-76 mutant in strain TUV93-0	John Leong
TUV93-0 ΔOI-102 (Cam^R)	Lambda red generated OI-102 mutant in strain TUV93-0	John Leong
TUV93-0 ΔOI-112-113 (Cam^R)	Lambda red generated OI-112-113 mutant in strain TUV93-0	John Leong
TUV93-0 ΔOI-133 (Cam^R)	Lambda red generated OI-133 mutant in strain TUV93-0	John Leong
TUV93-0 ΔOI-138 (Cam^R)	Lambda red generated OI-138 mutant in strain TUV93-0	John Leong
TUV93-0 ΔOI-139 (Cam^R)	Lambda red generated OI-139 mutant in strain TUV93-0	John Leong
TUV93-0 ΔOI-141 (Cam^R)	Lambda red generated OI-141 mutant in strain TUV93-0	John Leong
TUV93-0 ΔOI-153 (Cam^R)	Lambda red generated OI-153 mutant in strain TUV93-0	John Leong
TUV93-0 ΔOI-154 (Cam^R)	Lambda red generated OI-154 mutant in strain TUV93-0	John Leong
TUV93-0 ΔOI-156 (Cam^R)	Lambda red generated OI-156 mutant in strain TUV93-0	John Leong
TUV93-0 ΔOI-172 (Cam^R)	Lambda red generated OI-172 mutant in strain TUV93-0	John Leong
TUV93-0 ΔOI-173-175 (Cam^R)	Lambda red generated OI-173-175 mutant in strain TUV93-0	John Leong
TUV93-0 Δ*ecs1581* (Kan^R)	Allelic exchange strain with a kanamycin resistance cassette inserted into the open reading frame of *ecs1581* in strain TUV93-0	This study

TUV93-0 Δ*qseC* (Kan^R)	Allelic exchange strain with a kanamycin resistance cassette inserted into the open reading frame of *qseC* in strain TUV93-0	This study
CFT073	UPEC pyelonephritis strain CFT073 (serotype O6:K2:H1)	Mobley *et al*.,1990
CFT073 Δ*c1493* (Kan^R)	Allelic exchange strain with a kanamycin resistance cassette inserted into the open reading frame of *c1493* in strain CFT073	This study
ZAP198<br>ZAP198 Δ*grlA*::Tn5 (Kan^R)	Wild type EHEC O157:H7 strain<br>*grlA* transposon mutant in EHEC strain ZAP198 (Tn5)	Low *et al*., 2006<br>This study
ZAP1327 (Kan^R)	Constitutive LEE1 expression strain with a kanamycin resistance cassette inserted into the LEE1 promoter region in strain ZAP198	This study
ZAP1004	Unmarked *ler* deletion mutant in strain ZAP198	Low *et al*., 2006
ED1a	Commensal *E. coli* strain (serotype O81) isolated from the faeces of a healthy individual	Clermont *et al*., 2008
DH5α	Host strain used for cloning with pWSK29	Invitrogen
BL21 (DE3)	Bacteriophage T7 expression *E. coli* strain used with pET28a (+) to make recombinant His-tagged proteins	Invitrogen
**Plasmids**		
**Cloning plasmids**		
pET28a (+) (Kan^R)	His-tagged expression vector	Novagen
pKC26 (Cam^R)	Promoter-less GFP+ reporter fusion construct	Holden *et al*., 2007
pWSK29/pWControl (Amp^R)	IPTG inducible low copy number cloning vector	Wang and Kushner, 1991
pIB073 (Cam^R)	Chloramphenicol resistant temperature sensitive plasmid used for allelic exchange	Blomfield *et al*, 2001
pDG28 (Cam^R Kan^R)	Host vector harbouring the kanamycin resistance cassette used for all pIB073 based allelic exchanges	Gally *et al*., 1994
**Complementation plasmids**<br>pZ1835-Z1843	 *z1835-z1843* cloned into low copy number plasmid pWSK29	 This study
pECs1581/pECs1581 (LI)	*ecs1581* amplified from strain TUV93-0 and cloned into pWSK29 (representative of Lineage I EHEC O157:H7 strain variants)	This study
pTW14539 (LI/II)	EHEC *ecs1581* orthologue (*ecsp_1496*) amplified from strain TW14539 and cloned into pWSK29 (representative of Lineage I/II and	This study

	II O157:H7 strain variants)	
p96788 (LII)	EHEC *ecs1581* orthologue amplified from strain 96788 (un-sequenced) and cloned into pWSK29 (representative of Lineage II and LI/II O157:H7 strain variants)	This study
pC1493	*c1493* cloned into pWSK29	This study
pECED1_1787	*eced1_1787* cloned into pWSK29	This study
pQseC	*qseC* cloned into pWSK29	This study
pECED1_1787C20R	*eced1_1787* cloned into pWSK29 with a Cys to Arg substitution at position 20	This study
pECED1_1787C20R+N35K	*eced1_1787* cloned into pWSK29 with a Cys to Arg and Asn to Lys substitution at positions 20 and 35 respectively	This study
pECs1581R20C	*ecs1581* cloned into pWSK29 with an Arg to Cys substitution at position 20	This study
pECs1581R20C20N	*ecs1581*R20C cloned into pWSK29 with an Arg to Asn substitution at position 20	This study
pECs1581K35N	*ecs1581* cloned into pWSK29 with a Lys to Asn substitution at position 35	This study
pECs1581R20C+D10A	*ecs1581* cloned into pWSK29 with an Arg to Cys and Asp to Ala substitution at positions 20 and 10 respectively	This study
pECs1581R20C+K35N	*ecs1581* cloned into pWSK29 with an Arg to Cys and Lys to Asn substitution at positions 20 and 35 respectively	This study
pECs1581R20C+Q27K	*ecs1581* cloned into pWSK29 with an Arg to Cys and Gln to Lys substitution at positions 20 and 27 respectively	This study
pECs1581R20C+H45R	*ecs1581* cloned into pWSK29 with an Arg to Cys and His to Arg substitution at positions 20 and 45 respectively	This study
pECs1581R20C+R61D	*ecs1581* cloned into pWSK29 with an Arg to Cys and Arg to Asp substitution at positions 20 and 61 respectively	This study
**Recombinant protein expression**		
pET28a-ECs1581	*ecs1581* cloned into expression vector pET28a (+)	This study
pET28a-ECED1_1787	*eced1_1787* cloned into expression vector pET28a (+)	This study
**Transcriptional fusion (GFP+)**		

pLEE1-GFP	Upstream region of LEE1 cloned into GFP+ reporter pKC26 to create transcriptional fusion construct pLEE1-GFP	This study
**Allelic exchange**		
pIBECs1581-US<br>pIBECs1581-DS<br>pIBECs1581- Kan^R	Upstream flanking region of *ecs1581* cloned into pIB073<br>Downstream flanking region of *ecs1581* cloned into pIB073<br>Kanamycin resistance cassette cloned in between *ecs1581* US and DS flanking regions on pIB073	This study
pIBC1493-US<br>pIBC1493-DS<br>pIBC1493-Kan^R	Upstream flanking region of *c1493* cloned into pIB073<br>Downstream flanking region of *c1493* cloned into pIB073<br>Kanamycin resistance cassette cloned in between *c1493* US and DS flanking regions on pIB073	This study
pIBQseC-US<br>pIBQseC-DS<br>pIBQseC-Kan^R	Upstream flanking region of *qseC* cloned into pIB073<br>Downstream flanking region of *qseC* cloned into pIB073<br>Kanamycin resistance cassette cloned in between *qseC* US and DS flanking regions on pIB073	This study
**Primers**<br>**Plasmid complementation**		
z1835-z1843.F<br>z1835-z1843.R<br>ecs1581.F<br>ecs1581.R	CGTCTAGACTGCAACCGATTATAACGGATGCTTAACG<br>GGAAGCTTTTACGCATGACTGTCCGGTGTTACTTCAG<br>CGTCTAGACTTATCAGGTCACGCTATCG<br>CGAAGCTTATCACCTCTGTGTCTTTGTG	This study<br><br>This study
c1493.F<br>c1493.R	CGTCTAGATGAGTTGCAGGAAATAGAGA<br>CGAAGCTT CGTCATGTTCCTGCCATTG	This study
eced1_1787.F<br>eced1_1787.R	CGAAGCTTTTACCTCTGTGTCTTTGTG<br>CGTCTAGAGCTTATCAGGTCACGCTATC	This study
TW14539/96788.F<br>TW14539/96788.R	CGTCTAGAAGAACAGCACACCGGAGAAT<br>CGAAGCTTATGTGCCCAGGCATCACTAC	This study
ler.F<br>ler.R	AAAAACTGCAGGTATCATATAGCATCATATAGTG<br>AAAAAGTCGACTCATGTTAAATATTTTTCAGCGG	This study
eced1_1787C20R.F<br><br>eced1_1787C20R.R	CGAAGCTTTTACCTCTGTGTCTTTGTGTTTTATTCCGCGTGGCT<br>GTTTTTTTACGTTCGGTGCCAGTTTCATTTCTTACCCCTGAATACG<br>CGTCTAGACTATCGGGGAATAAAAATAATGAAACTTAAATAT	This study<br><br>This study

	CCTGGCTTAACTGCCAGCGGCAAAACTCGCACTAAATTCATG<br>CGCGGG	
eced1_1787C20R+N35K.F<br>eced1_1787C20R+N35K.nestR<br>eced1_1787C20R+N35K.nestF<br>eced1_1787C20R+N35K.R	CGAAGCTTCAGCTATGACCATGTATTACG<br>TTCCTCCACGCCCTTAATCAT<br>ATGATTAAGGGCGTGGAGGAA<br>CGTCTAGACTGTGTCTTTGTGTTTTATTC	This study
ecs1581R20C.F<br><br><br>ecs1581R20C.R	CGTCTAGACTATCGGGGAATAAAAATAATGAAACTTAAATAT<br>CCTGGCTTAACTGACAGCGGCAAAACTCGCACTAAATTCATG<br>TGCGGG<br>CGAAGCTTATCACCTCTGTGTCTTTGTGTTTTATTCCGCGTGGC<br>TGTTTTTTTACGTTCGGTGCCAGTTTCATTTTTTACCCCTGAATAC	This study
ecs1581R20C20N.F<br>ecs1581R20C20N.nestR<br>ecs1581R20C20N.nestF<br>ecs1581R20C20N.R	CGTCTAGACGCTATGACCATGATTACGC<br>GTAAATATCCCCGTTCATGAA<br>TTCATGAACGGGGATATTTAC<br>CGAAGCTTATCACCTCTGTGTCTTTGTG	This study
ecs1581K35N.F<br>ecs1581K35N.nestR<br>ecs1581K35N.nestF<br>ecs1581K35N.R	CGTCTAGACTTATCAGGTCACGCTATCG<br>TTCCTCCACGCCATTAATCAT<br>ATGATTAATGGCGTGGAGGAA<br>CGAAGCTTATCACCTCTGTGTCTTTGTG	This study
ecs1581R20C+D10A.F<br><br>ecs1581R20C+D10A.R	CGTCTAGACTATCGGGGAATAAAAATAATGAAACTTAAATAT<br>CCTGGCTTAACTGCCAGCGGC<br>CGAAGCTTCACCTCTGTGTCTTTGTGTTTTATTCCGCGTGGCTG<br>TTTTTTTACGTTCGGTGC	This study
ecs1581R20C+K35N.F<br>ecs1581R20C+K35N.nestR<br>ecs1581R20C+K35N.nestF<br>ecs1581R20C+K35N.R	CGTCTAGACGCTATGACCATGATTACGC<br>TTCCTCCACGCCATTAATCAT<br>ATGATTAATGGCGTGGAGGAA<br>CGAAGCTTATCACCTCTGTGTCTTTGTG	This study
ecs1581R20C+H45R.F<br>ecs1581R20C+H45R.nestR<br>ecs1581R20C+H45R.nestF<br>ecs1581R20C+H45R.R	CGTCTAGACGCTATGACCATGATTACGC<br>GTAGCCTTCACGGCGGTAAGT<br>ACTTACCGCCGTGAAGGCTAC<br>CGAAGCTTATCACCTCTGTGTCTTTGTG	This study
ecs1581R20C+R61D.F<br>ecs1581R20C+R61D.nestR	CGTCTAGACGCTATGACCATGATTACGC<br>AGAAAAATCACGGTCGAACTG	This study

ecs1581R20C+R61D.nestF<br>ecs1581R20C+R61D.R	CAGTTCGACCGTGATTTTTCT<br>CGAAGCTTATCACCTCTGTGTCTTTGTG	
ecs1581R20C+Q27K.F<br>ecs1581R20C+Q27K.nestR<br>ecs1581R20C+Q27K.nestF<br>ecs1581R20C+Q27K.R	CGTCTAGACGCTATGACCATGATTACGC<br>CGTGCCGCCGTACTTATCGCG<br>CGCGATAAGTACGGCGGCACG<br>CGAAGCTTATCACCTCTGTGTCTTTGTG	This study
**Recombinant protein expression**		
ecs1581-pET28a.F<br>ecs1581-pET28a.R<br>eced1_1787-pET28a.F<br>eced1_1787-pET28a.R<br>eced1_1787C20R-pET28a.F<br>eced1_1787C20R-pET28a.R<br><br>**EMSA**<br>EMSA-LEE1.F<br>EMSA-LEE1.R<br>EMSA-gapA.F<br>EMSA-gapA.R	CGGGATCCATGAAACTTAAATATCCTGG<br>GGGTCGACTACGTTCGGTGCCAGTTTCA<br>AAGTCGACTTACGTTCGGTGCCAGTTTCA<br>AAGGATCCATGAAACTTAAATATCCTGGC<br>AAGTCGACTTACGTCGGTGCCAGTTTCA<br>AAGGATCCATGAAACTTAAATATCCTGGC<br><br><br>CATGCTTTAATATTTTAAGCTATTAGCGAC<br>GCAATGAGATCTATCTTATAAAGAGAAACG<br>ACATTAACACCAACTGGCAAAATTTTGTCC<br>CATATATTCCACCAGCTATTTGTTAGTG	This study<br><br>This study<br><br>This study<br><br><br><br>This study<br><br>This study
**RT-PCR**<br>RT-ler.F<br>RT-ler.R<br>RT-16S.F<br>RT-16S.R	 CTGCGAGAGCAGGAAGTTCA<br>AGGCACATTAGTATATCCCAGCTC<br>ATTGACGTTACCCGCAGAAG<br>CGCTTTACGCCCAGTAATTC	 This study

**Screening and sequencing**		
pWSK29-screen.F	CAGTCACGACGTTGTAAAAC	This study
pWSK29-screen.R	CGTATGTTGTGTGGAATTGT	
pIB073-screen.F	CCTGTCCTACGAGTTGCATG	This study
pIB073-screen.R	GACTCCTGCATTAGGAAGCA	
pET28a-screen.F	CTTTGTTAGCAGCCGGATCTC	This study
pET28a-screen.R	CATCATCATCATCACAGCAGC	
pKC26-screen.F	GATGCAATTTCTATGCGCACC	This study
pKC26-screen.R	GTGAAGGTGATGCTACATACG	

Table S2. List of ECs1581 orthologous and paralogous proteins present in *E. coli* and Shigella *spp* strains

ECs1581 variant (pathotype/strain/locus tag)	Length (aa)	Residues found at positions 19-22	% sequence similarity with ECs1581/ overlap region (aa)	Comments/references
**EHEC strains Sakai/EDL933**				
ECs1581	99	MRGD	100 over 99	These prototypical EHEC strains were isolated during large outbreaks in Japan and the US from radish sprouts and undercooked hamburger meat respectively. Perna *et al*., 2001; Hayashi *et al*., 2001
ECs5415 (SpLE1)	63	GALV	39 over 41	
Z1197 (OI-43)	63	GALV	39 over 41	
Z1636 (OI-48)	63	GALV	39 over 41	
**Commensal *E. coli* strain ED1a (O81)**				
ECED1_1787	99	MCGD	94 over 99	Commensal *E. coli* strain isolated from the faeces of a healthy individual. Clermont *et al*., 2008
ECED1_ 2647	99	IPGA	31 over 99	
ECED1_3312	97	MRGD	67 over 97	
ECED1_3445	63	GALA	42 over 45	
**Atypical EPEC strain E110019**				
EcE110019_0327	99	MRGD	92 over 99	Atypical EPEC strain E110010 was isolated from an outbreak in a school in Finland. Unlike most EPEC, E110019 spread from person-to-person to over 100 close contacts out with the school. This strain lacks the EAF plasmid. Rasko *et al*., 2008, Craig. J. Venter Institute (unpublished)
EcE110019_4878	99	TRGD	80 over 99	
***Shigella flexneri* 5 strain 8401**				
SFV_1149	99	TRGD	88 over 99	Nie *et al*., 2006

***Shigella flexneri* strain 2002017**				
SFxv_1290	99	TRGD	88 over 99	*S. flexneri* 2002017 is an emerging multi drug resistant serotype X variant of Shigella, isolated in China from an epidemic clone. Ye *et al*., 2010
***Shigella dysenteriae* strain 1012**				
Sd1012_1853	99	MNGD	85 over 99	*S. dysenteriae* 1012 was isolated in Bangladesh and is representative of type 4 *S. dysenteriae* that are becoming more prevalent in humans infections. Studies have shown this to be one of the most virulent *S. dysenteriae* strains identified Rasko *et al*., 2008, J. Craig.Venter Institute
Sd1012_2233	99	MRGD	79 over 99	
***E. coli* sp 3_2 53FAA**				
ESAG_00330	99	MNGD	84 over 99	*E. coli sp.* 3_2_53FAA was isolated from an intestinal biopsy specimen taken from the descending colon of a 52-year old male with active Crohn's disease.
**NMEC *E. coli* strain IHE3034**				
ECOK1_0888	99	MNGD	84 over 99	Recently sequenced newborn meningitis *E. coli* strain. Moriel *et al.,* 2010
**Adherent invasive *E. coli* strain UM146**				
UM146_13215	99	MNGD	84 over 99	Strain UM146 was isolated from an ileal Crohn's disease biopsy tissue.

**UPEC strain CFT073**				
C1493	99	MRGD	83 over 99	Prototypical UPEC *E. coli* strain CFT073. Mobley *et al*., 1990
C4520	63	GALA	42 over 41	
**UPEC strain IAI39**				
ECIAI39_2021	99	MNGD	83 over 99	Touchon *et al*., 2009
ECIAI39_0509	95	SLGD	44 over 92	
**EHEC O26:H11 strain 11368**				
ECO26_1651	99	TRGD	81 over 99	Ogura *et al*., 2009
ECO26_3111	99	TRGD	78 over 99	
ECO26_2322	99	IPGA	29 over 99	
ECO26_ 1322	63	GALV	39 over 41	
**EHEC strain TW14539**				
ECSP_1496	99	TRGD	81 over 99	A spinach outbreak isolate of EHEC O157:H7 that is considered to be hyper-virulent. Abu-Ali *et al*., 2010
ECSP_1303	63	GALV	39 over 41	
**EHEC O103:H2 strain 12009**				
ECO103_1241	99	TRGD	81 over 99	Ogura *et al*., 2009
**EPEC O55:H7 CB9615**				
G2583_1395	99	TRGD	81 over 99	This strain is considered to be the closest ancestor of EHEC O157:H7 clones. Zhou *et al*., 2010
**EAEC O42**				
EC042_1760	99	TRGD	80 over 99	Prototypical enteroaggregative *E. coli* strain O42. Chaudhuri *et al*., 2010
EC042_2163	99	RPGD	68 over 99	
**EHEC O111:H- strain 11128**				
ECO111_2919	99	TRGD	78 over 99	Ogura *et al*., 2009

Figure S1

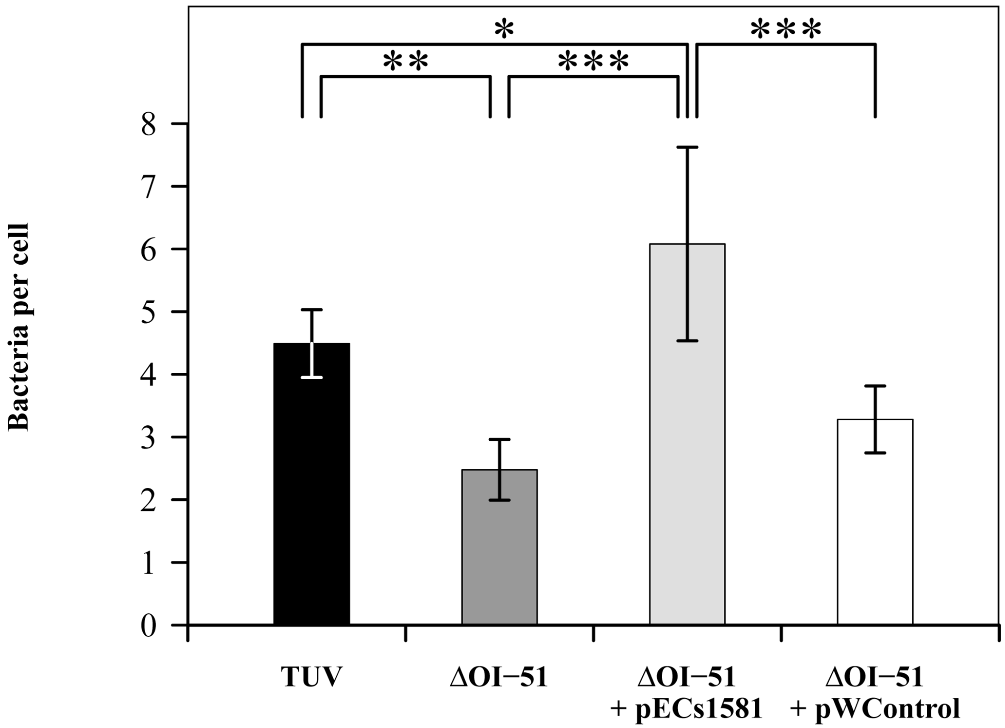

Fig. S1. Analysis of bacterial adherence to bovine epithelial cells. Adherence assays were carried out as defined in Experimental Procedures and the data shown represents the binding of bacteria per epithelial cell. ***P = <0.001. ** P = 0.005. * P = 0.035.

Figure S2

```
                                         Ler linker region                 Ler/H-NS DNA binding motif
Ler        1 MRRLFIMNMENNSHTTSPYIQLIEQIAVLQQEAKRLREQEVQSVIESIQK     50

ECs1581    0 --------------------------------------------------      0

Ler       51 QITYYNITLQELGYTNVPDDGLARRNSSKGVYYRNEEGQTWSGVGRQPRW    100
                      .:|.|..:.|.|..|....:|..||::.|.|
ECs1581    1 ---------MKLKYPGLTDSGKTRTKFMRGDIYRDQYGGT----------     31

Ler      101 LKEALLNGMKKEDFLVKDTEEEIIPLKNI---------------------    129
                          .::|..||..:.....
ECs1581   32 -------------VMIKGVEERRVTYHREGYEYDCVMPVYQFRRDFSLVQ     68

Ler      129 -------------------------------    129

ECs1581   69 AAPRSKPTSREKARANIQEIKKMLNVFRGKK     99
```

Fig. S2. Alignment of Ler and ECs1581 amino acid sequences showing slight homology between the two proteins at the N-terminus of ECs1581 and the linker/oligomerisation region of Ler. The established DNA binding motif and linker region of Ler are annotated (Mellies *et al*., 2008).

Figure S3

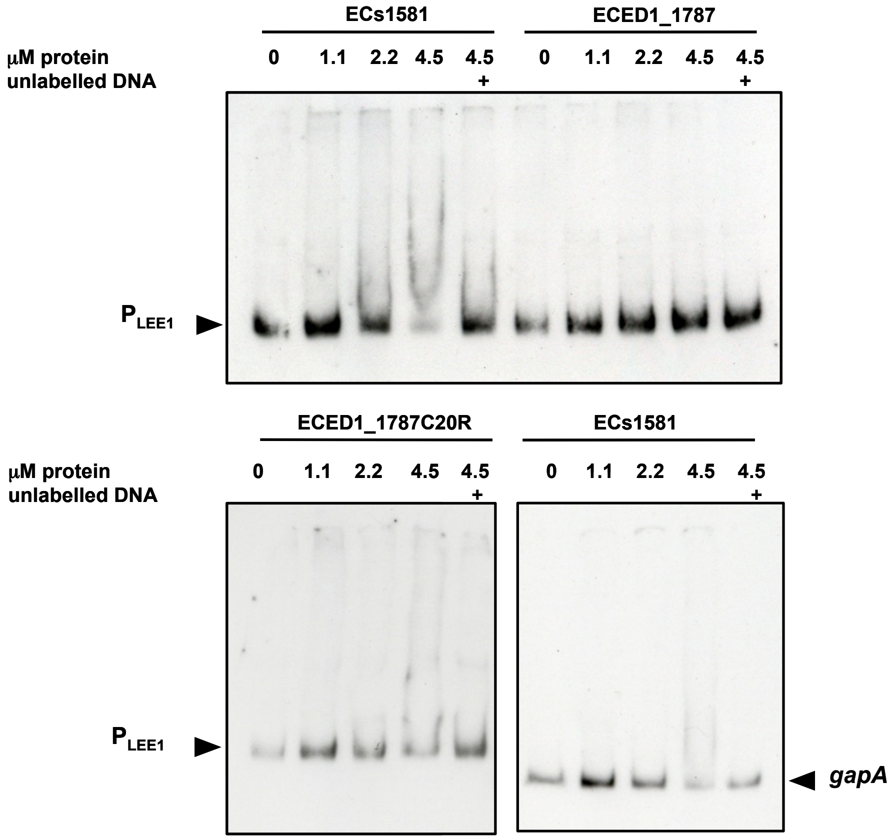

Fig. S3. Electrophoresis mobility shift assays examining ECs1581, ECED1_1787 and ECED1_1787C20R binding to specific DNA regions. Purified ECs1581, ECED1_1787 and ECED1_1787C20R were added to the LEE1 promoter. As a control for non-specific interactions, ECs1581 was added to a *gapA* control fragment at the concentrations shown. EMSAs were carried out as defined in Experimental Procedures. While ECs1581 did interact with the LEE1 promoter region this binding was relatively non-specific. By contrast, the ECED1_1787 variant demonstrated no interaction with the LEE1 promoter region at the same concentrations. The ECED1_1787C20R showed some interaction which was reversed by addition of excess unlabelled LEE1 DNA.

Figure S4

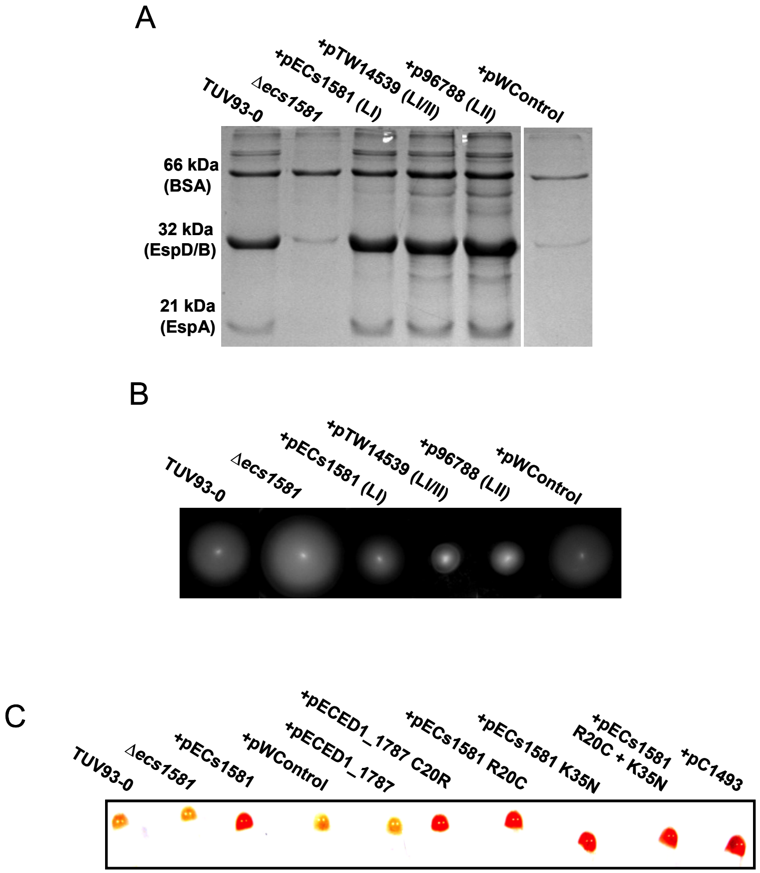

Fig. S4. (A) SDS-PAGE gel showing T3S levels in strain TUV93-0 and Δ*ecs1581* mutant expressing EHEC O157:H7 lineage specific variants of ECs1581 from a low copy number plasmid (pECs1581/LI, pTW14539/LI/II, p96788/LII) and pWControl. (B) Analysis of motility in strain TUV93-0 and Δ*ecs1581* mutant expressing pECs1581/LI, pTW14539/LI/II, p96788/LII or pWControl. (C) Congo-red binding capacity of a selection of natural and engineered variants (pECs1581, pWControl, pECED1_1787, pECED1_1787C20R, pECs1581K35N, pECs1581R20C+K35N and pC1493). Analysis of culture supernatants, motility and Congo-red binding was carried out as described in the Experimental Procedures.
